# Breastfeeding reduces ultra-processed foods and sweetened beverages consumption among children under two years old

**DOI:** 10.1186/s12889-020-8405-6

**Published:** 2020-03-14

**Authors:** Ana Maria Spaniol, Teresa Helena Macedo da Costa, Gisele Ane Bortolini, Muriel Bauermann Gubert

**Affiliations:** 1 Postgraduate Program in Human Nutrition, Center for Epidemiological Studies in Health and Nutrition – NESNUT, University of Brasilia, Brasilia, Federal District Brazil; 2 Department of Nutrition, University of Brasilia, Brasilia, Federal District Brazil; 3 General Coordination of Food and Nutrition, Ministry of Health, Brasília, Federal District Brazil; 4 Department of Nutrition, Center for Epidemiological Studies in Health and Nutrition – NESNUT, University of Brasilia, Brasilia, Federal District Brazil

**Keywords:** Children, Primary health care, Breastfeeding, Complementary feeding, Ultra-processed foods, Sweetened beverages

## Abstract

**Background:**

Breastfeeding and adequate complementary feeding are associated with healthy eating habits, prevention of nutritional deficiencies, obesity and non-communicable diseases. Our aim was to identify feeding practices and to evaluate the association between breastmilk intake and complementary feeding, focusing on ultra-processed foods (UPF) and sweetened beverages, among children under 2 years old.

**Methods:**

We conducted a cross-sectional study including 847 children from 20 Primary Health Units. We evaluated children’s food consumption using a food intake markers questionnaire. We conducted a logistic regression to evaluate the effect of breastmilk intake on feeding practices.

**Results:**

The breastmilk intake was associated with lower odds of consuming non-recommended foods, such as cookies or crackers (OR: 0.29; IC 95%: 0.20–0.41) for children under 6 months, yogurt (OR: 0.33; CI 95%: 0.12–0.88) for children between 6 and 12 months and soft drinks (OR: 0.36; CI 95%: 0.17–0.75) for children between 12 and 24 months. Moreover, the breastmilk intake was associated with lower odds of consuming UPF (OR: 0.26; CI 95%: 0.09–0.74) and sweetened beverages (OR: 0.13; CI 95%: 0.05–0.33) for children under 6 months. For children between 12 and 24 months, breastmilk intake was associated with lower odds of consuming sweetened beverages (OR: 0.40; CI 95%: 0.24–0.65).

**Conclusion:**

Breastmilk intake was associated with a reduced consumption of UPF and sweetened beverages. Investment in actions to scale up breastfeeding can generate benefits, besides those of breastmilk itself, translating into better feeding habits and preventing health problems in childhood.

## Background

During the first 1000 days of life following birth, healthy feeding practices are associated with the reduction of morbidity and mortality in childhood [1]. During the first 6 months of life, breastmilk is enough to supply all of the child’s nutritional requirements and after this period, breastfeeding, along with opportune and adequate complementary feeding, contribute to establishing healthy eating habits and are related to repercussions for lifetime health [2–7].

Optimal breastfeeding rates correspond to exclusive breastfeeding until the sixth month of life and to continued breastfeeding up to 2 years of age, to contribute to achieve optimal growth, development and health, according to the recommendations of the World Health Organization [8]. Thus, optimal breastfeeding and healthy complementary feeding are associated with social, economic and familial factors and still a challenge in high and middle-income countries, especially regarding the avoidance of early and high consumption of ultra-processed foods (UPF) [2, 9, 10]. UPF usually present high energy density and low nutritional quality, and should not be consumed by children under 2 years old, since they are associated with overweight, chronic diseases and micronutrient deficiencies [11–13].

The breastmilk intake, in addition to its own benefits to health [2], is also related to healthier feeding practices in childhood, such as a timely food introduction, more diversified diets and a lower consumption of unhealthy foods among children [14–18]. In Brazil, there is a lack of studies exploring this topic, especially regarding the association between breastfeeding and UPF consumption before 2 years of age, a crucial period to establish long term healthy dietary patterns [19, 20]. Thus, the aim of our study was to identify feeding practices and to evaluate the association between breastmilk intake and complementary feeding, focusing on the UPF and sweetened beverages consumption, among children under 2 years old.

## Methods

### Design and population of the study

We conducted a cross-sectional study in Primary Health Units (PHU) in Federal District, Brazil. To calculate the sample size, we assumed a margin of error of 5% and a confidence level of 97%, based on the prevalence of 50% of exclusive breastfeeding among children under 6 months in Federal District [21]. We also considered the number of children under 2 years old attended in PHU in 2015 and adopted a two-stage sampling, listing all the local PHU (*n* = 131) and randomly selecting 20 PHU. Based on the number of children attending in each selected PHU, we define the proportional sample. The minimum sample size was determined to be 520 children, considering an attrition rate of 10%. The final sample recruited was 847 children.

We included all children under 2 years old on the date of interview accompanied by their mothers, and excluded twin and adopted children.

### Data collection and measures

Data collection was conducted from March 2017 to March 2018. All children under 2 years old who attended the PHU for childcare follow-up consults were invited to participate.

The questionnaire was applied by trained interviewers, answered by the child’s mother and was comprised of socioeconomic and demographic questions relating to the mother, family and household, household food insecurity situation, prenatal, puerperium, child birth, health and food consumption.

We evaluated children’s food consumption using an adapted version of the Food Intake Markers Questionnaire proposed by Brazilian Ministry of Health [22]. This tool consists of “yes” or “no” questions about consumption of foods items or groups related to the day before the interview. The original tool is composed by two sets of questions: one for children under 6 months and another one for children between 6 and 24 months of age. The set of questions for children under 6 months of age is limited and include questions about the consumption of breastmilk and foods and beverages that are usually offered before 6 months of life causing exclusive breastfeeding interruption, such as water, tea and infant formula. Thus, this set of questions only allows to calculate the “exclusive breastfeeding” dietary indicator. The set of questions for children aged between 6 and 24 months is more comprehensive and includes questions about child’s consumption of food groups and ultra-processed foods, such as: breastmilk, fruit, salty food, meats or eggs, processed juice, soft drink, yogurt and packaged snacks. It also asks about frequency of fruit consumption and about frequency and consistency of salty food consumption. Therefore, this set of questions allows the calculation of different dietary indicators, described below. In our study, we chose to apply the two set of questions to all children, in order to be able to calculate the dietary indicators mentioned below, no matter child’s age.

We performed the Food Intake Markers analysis according to the recommendations of the Ministry of Health and calculated seven indicators: exclusive breastfeeding, continued breastfeeding, minimum dietary diversity, minimum meal frequency and proper consistency, iron-rich foods and vitamin-A-rich foods consumption, sweetened beverages consumption and UPF consumption [22]. To calculate the minimum dietary diversity indicator, we did not consider yogurt consumption because although this food constitutes a dairy products group in the original protocol, it commonly presents high levels of sugars, flavours and other additives [23]. Thus, we considered yogurt as an UPF [11, 12]. Finally, to calculate the UPF consumption indicator we considered the proportion of children who consumed at least one type of UPF at the day before, such as: hamburger or processed meats; sweetened beverages, including soft drinks, processed juice, food or beverage with added sugar, honey or artificial sweetener; yogurt; instant noodles; packaged snacks; processed crackers or cookies; or confectionery.

We evaluated household food security using the Brazilian Household Food Insecurity Measurement Scale (EBIA), classifying the households as experiencing secure, mild, moderate or severe food insecurity [24].

To control the quality of the data collection, we reapplied three questions from different parts of the questionnaire by telephone until 2 weeks after the interview to 20% of the total sample of mothers from each PHU.

### Statistical analyses

We conducted the analyses in the Statistical Package for The Social Sciences (SPSS), version 20.0. First, we performed an exploratory analysis and eliminated 39 cases with more than 10% of unanswered variables. None of the variables presented in this analysis include more than 10% of missing data.

Subsequently, we conducted a prevalence analysis, with socioeconomic, demographic and health variables. We also presented the prevalence of children’s food consumption variables stratified by age group (children under 6 months, children between 6 and 12 months and children between 12 and 24 months). It is important to note that, when analyzing the food consumption of children under 6 months of age, we excluded those who were exclusively breastfed (*n* = 325) and we only evaluated the consumption of non-recommended foods, since they should not ideally receive any food other than breastmilk [3].

Next, we performed the Pearson’s chi-square test to verify the association between breastmilk intake and food consumption and feeding indicators. We performed crude and adjusted logistic regressions, considering breastmilk intake as independent variable, for foods that presented *p* < 0.20 in chi-square test and for all feeding practices indicators. For adjusted analysis, we considered as confounding variables: maternal education (as socioeconomic status proxy), household food security, work situation, mother’s marital status and age group, number of prenatal consultations, type of child birth, parity, child’s gender, use of pacifier, bottle feeding and child hospitalization in last year. The confounding variables with *p* < 0.20 remained in final model. The variables that did not present sufficient cases (*n* = 0 in at least one of the table cells) to perform the logistic regression were excluded from the analyses (soft drinks and processed meats for children under 6 months and processed juice and packaged snacks for children between 6 and 12 months).

## Results

Data from 847 children under 2 years of age were analyzed. The majority of the mothers had low schooling (80.8% had up to 8 years of schooling). The mothers’ average age was 27 years old (SD: 6.7; minimum 16.0; maximum 48.8 years) (data not presented in table). Follow-up prenatal care was attended by 98.8% of the mothers (data not presented in table), and of these, 90.0% had 6 or more prenatal consultations and 93.0% had prenatal consultations prior 20 gestational weeks. Only 23.8% of children received breastmilk in the first hour of life. The use of pacifiers and bottle-feeding on the day before the interview was of 32.3 and 58.9%, respectively (Table 1).

**Table 1:** Population descriptive characteristics. Federal District, Brazil, 2017–2018

Variable of study	*n*^a^	%
**Socioeconomic and demographic variables of the mother, family and household**		
**Mother’s Age Group**		
Under 20 years	112	13.3
20 years or more	731	86.7
**Mother’s Color**		
White	124	14.9
Black or brown	653	78.3
Yellow or indigenous	57	6.8
**Marital Status**		
Lives with partner	560	66.6
Does not live with partner	281	33.4
**Maternal education**		
Up to 4 years	193	22.9
Between 4 to 8 years	487	57.9
Between 8 to 12 years	73	8.7
Over 12 years	88	10.5
**Household Food and Nutritional Security**		
Food Security	494	59.4
Mild food insecurity	255	30.6
Moderate food insecurity	53	6.4
Severe food insecurity	30	3.6
**Work situation**		
Working outside the home	176	21.4
On maternity leave	56	6.8
Not working outside the home	589	71.7
**Prenatal, delivery and puerperium conditions**		
**Number of prenatal consultations**		
Less than 6	82	10.0
6 or more	742	90.0
**Gestational weeks for first prenatal consultation**		
Less than 20 gestational weeks	784	93.0
20 gestational weeks or more	59	7.0
**Type of child birth**		
Normal	468	55.5
Cesarean	375	44.5
**Primiparity**		
Yes	412	48.9
No	431	51.1
**Characteristics of the children**		
**Children age group**		
Under 6 months	202	23.8
Between 6 and 12 months	208	24.6
Between 12 and 24 months	437	51.6
**Child’s gender**		
Male	430	51.0
Female	413	49.0
**Child’s color**		
White	318	38.4
Black or brown	459	55.4
Yellow or indigenous	52	6.3
**Breastfeeding in the first hour of life**		
Yes	200	23.8
No	641	76.2
**Use of pacifiers the previous day**		
Yes	271	32.3
No	568	67.7
**Bottle feeding the previous day**		
Yes	496	58.9
No	346	41.1

^a^The total was lower for some variables due to missing information

Table 2 presented the foods consumed and the feeding indicators by age group. Among children under 6 months who were not exclusively breastfed, 82.7% received breastmilk the day before interview. Still, 23.3% consumed at least one UPF the previous day, with highest prevalence of foods or beverages with added sugar, honey o artificial sweetener (18.4%) and cookies or crackers (17.3%) consumption. Regarding children between 6 and 12 months, only 33.7% presented minimum dietary diversity and 56.3% consumed at least one UPF. For children between 12 and 24 months, only 44.6% presented minimum dietary diversity and 86.3% consumed UPF, especially yogurt (40.3%), food or beverage with added sugar, honey or artificial sweetener (48.2%) and cookies or crackers (70.0%). A high prevalence of children between 6 and 12 months and between 12 and 24 months consumed iron-rich foods (49.0 and 74.1%) and vitamin-A-rich foods (65.4 and 63.2%), respectively.

**Table 2:** Prevalence of food consumption on the day before the interview and feeding practices indicators according to children age group. Federal District, Brazil, 2017–2018

Food consumed the day before the interview and feeding practices indicators	Children age group					
	Under 6 months		Between 6 and 12 months		Between 12 and 24 months	
	*n*	%	*n*	%	*n*	%
**Food consumed the day before the interview**						
Breastmilk	167	82.7	170	81.7	265	60.8
Processed juice	9	4.5	9	4.3	95	21.7
Soft drink	1	0.5	5	2.4	39	8.9
Yogurt	19	9.5	46	22.1	174	40.3
Processed meat	0	0.0	6	2.9	29	6.7
Instant noodles	8	4.0	7	3.4	31	7.2
Food or beverage with added sugar, honey or artificial sweetener	37	18.4	53	25.9	210	48.2
Confectionery	8	4.0	12	5.8	128	29.5
Cookies or crackers	35	17.3	84	40.6	304	70.0
Packaged snacks	5	2.5	9	4.3	53	12.2
**Feeding practices indicators**						
Minimum dietary diversity	–	–	70	33.7	195	44.6
Consumption of iron-rich foods	–	–	102	49.0	324	74.1
Consumption of foods high in vitamin A	–	–	136	65.4	276	63.2
Minimum frequency and adequate consistency	–	–	174	83.7	375	85.8
Consumption of ultra-processed foods	47	23.3	117	56.3	377	86.3
Consumption of sweetened beverages	41	20.3	62	29.8	256	58.6

The bivariate analysis showed that the breastmilk intake was not associated to the consumption of healthy foods.

The adjusted regression (Table 3) indicated that breastmilk intake was associated with lower odds of children under 6 months consuming foods or beverages with added sugar, honey or artificial sweetener (OR: 0.17; CI 95%: 0.06–0.49) and cookies or crackers (OR: 0.29; CI 95%: 0.20–0.41). Among children between 6 and 12 months, breastmilk intake was associated with lower odds of consuming yogurt (OR: 0.33; CI 95%: 0.12–0.88). Among children between 12 and 24 months, breastmilk intake was associated with lower odds of consuming processed juice (OR: 0.60; CI 95%: 0.37–0.97), soft drinks (OR: 0.36; CI 95%: 0.17–0.75%), foods or beverages with added sugar, honey or artificial sweetener (OR: 0.56; CI 95%: 0.35–0.91), and cookies or crackers (OR: 0.49; CI 95%: 0.30–0.80).

**Table 3:** Crude and adjusted odds ratio for food consumption according to breastmilk intake by children age group. Federal District, Brazil, 2017–2018

Food consumed the day before the interview	Children age group					
	Under 6 months		Between 6 and 12 months		Between 12 and 24 months	
	Crude OR (CI 95%)	Adjusted OR (CI 95%)	Crude OR (CI 95%)	Adjusted OR (CI 95%)	Crude OR (CI 95%)	Adjusted OR (CI 95%)
**Processed juice**						
Yes	0.40 (0.09–1.70)	0.24 (0.05–1.15)^a^	–	–	**0.61 (0.39–0.97)**	**0.60 (0.37–0.97)**^i^
No	1	1	–	–	1	1
**Soft drinks**						
Yes	–	–	–	–	**0.46. (0.23–0.89)**	**0.36 (0.17–0.75)**^j^
No	–	–	–	–	1	1
**Yogurt**						
Yes	–	–	**0.34 (0.16–0.73)**	**0.33 (0.12–0.88)**^f^	–	–
No	–	–	1	1	–	–
**Instant noodles**						
Yes	0.32 (0.07–1.44)	0.55 (0.29–1.05)^b^	0.28 (0.06–1.31)	0.28 (0.06–1.31)^g^	–	–
No	1	1	1	1	–	–
**Food or beverage with added sugar, honey or artificial sweetener**						
Yes	**0.28 (0.12–0.64)**	**0.17 (0.06–0.49)**^c^	–	–	0.68 (0.46–1.00)	**0.56 (0.35–0.91)**^k^
No	1	1	–	–	1	1
**Cookies or crackers**						
Yes	0.44 (0.18–1.02)	**0.29 (0.20–0.41)**^d^	0.62 (0.30–1.26)	0.64 (0.30–1.34)^h^	**0.51 (0.32–0.79)**	**0.49 (0.30–0.80)**^l^
No	1	1	1	1	1	1
**Packaged snacks**						
Yes	0.82 (0.08–7.57)	0.82 (0.08–7.57)^e^	–	–	0.57 (0.32–1.03)	0.58 (0.32–1.05)^m^
No	1	1	–	–	1	1
**Processed meat**						
Yes	–	–	–	–	1.73 (0.75–4.02)	2.06 (0.83–5.13)^n^
No	–	–	–	–	1	1

^a^Association adjusted for pacifier use the previous day

^b^Association adjusted for maternal education, mother’s marital status, number of prenatal consultations, primiparity and child’s gender

^c^Association adjusted for pacifier use the previous day, mother’s work situation and the child’s gender

^d^Association adjusted for maternal education, pacifier use the previous day, food and nutrition security situation and mother’s age group

^e^Association not presented confounding variables with *p* < 0.20

^f^Association adjusted for maternal education, mother’s marital status, number of prenatal consultations, type of child birth, mother’s age group and use of baby bottle the previous day

^g^Association not presented confounding variables with *p* < 0.20

^h^Association adjusted for number of prenatal consultations and mother’s work situation

^i^Association adjusted for number of prenatal consultations, mother’s age group, primiparity and hospitalization of the child in the last year

^j^Association adjusted for mother’s marital status and number of prenatal consultations

^k^Association adjusted for maternal education, household food security situation, mother’s work situation, mother’s age group, number of prenatal consultations, child’s gender, use of pacifier the previous day and hospitalization of the child in the last year

^l^Association adjusted for maternal education, household food security situation, mother’s work situation, number of prenatal consultations, type of child birth, primiparity and mother’s age group

^m^Association adjusted for type of child birth and hospitalization of the child in the last year

^n^Association adjusted for number of prenatal consultations, mother’s age group and mother’s work situation

Adjusted regression for feeding indicators (Table 4) showed no association between breastmilk intake and healthy feeding indicators. Among children under 6 months, breastmilk intake was associated with lower odds of consuming UPF (OR: 0.26; CI 95%: 0.09–0.74) and sweetened beverages (OR: 0.13; CI 95%: 0.05–0.33). Among children between 12 and 24 months, breastmilk intake was associated with lower odds of consuming sweetened beverages (OR: 0.40; CI 95%: 0.24–0.65).

**Table 4:** Crude and adjusted chance ratio for feeding practices indicators according to the breastmilk intake the day before the interview by children age group. Federal District, Brazil, 2017–2018

Feeding practices indicators	Children age group					
	Under 6 months		Between 6 and 12 months		Between 12 and 24 months	
	Crude OR (CI 95%)	Adjusted OR (CI 95%)	Crude OR (CI 95%)	Adjusted OR (CI 95%)	Crude OR (CI 95%)	Adjusted OR (CI 95%)
**Minimum dietary diversity**						
Yes	–	–	1.12 (0.52–2.38)	0.36 (0.12–1.05)^c^	0.95 (0.65–1.40)	0.85 (0.53–1.36)^h^
No	–	–	1	1	1	1
**Consumption of iron-rich foods**						
Yes	–	–	1.23 (0.60–2.50)	1.01 (0.43–2.39)^d^	0.76 (0.48–1.19)	0.66 (0.39–1.11)^i^
No	–	–	1	1	1	1
**Consumption of foods high in vitamin A**						
Yes	–	–	0.97 (0.46–2.05)	1.25 (0.47–3.30)^e^	0.93 (0.62–1.38)	1.04 (0.66–1.63)^j^
No	–	–	1	1	1	1
**Minimum frequency and adequate consistency**						
Yes	–	–	1.19 (0.47–2.99)	0.74 (0.26–2.10)^f^	1.33 (0.77–2.28)	1.32 (0.76–2.27)^k^
No	–	–	1	1	–	–
**Consumption of ultra-processed foods**						
Yes	0.50 (0.23–1.12)	**0.26 (0.09–0.74)**^a^	0.61 (0.29–1.27)	0.56 (0.23–1.35)^g^	0.62 (0.34–1.12)	0.60 (0.32–1.14)^l^
No	1	1	1	1	1	1
**Consumption of ssweetened beverages**						
Yes	**0.24 (0.11–0.54)**	**0.13 (0.05–0.33)**^b^	–	–	**0.55 (0.37–0.82)**	**0.40 (0.24–0.65)**^**m**^
No	1	1	–	–	1	1

^a^Association adjusted for use of pacifier the previous day and mother’s work situation

^b^Association adjusted for mother’s marital status and the use of pacifier the previous day

^c^Association adjusted for maternal education, mother’s marital status, number of prenatal consultations, gestational weeks for first prenatal consultation, household food security situation, mother’s work situation, use of baby bottle and hospitalization of the child in the last year

^d^Association adjusted for maternal education, mother’s work situation and use of pacifier the previous day

^e^Association adjusted for maternal education, number of prenatal consultations, use of pacifier the previous day and hospitalization of the child in the last year

^f^Association adjusted for maternal education, mother’s age group, use of baby bottle the previous day and hospitalization of the child in the last year

^g^Association adjusted for maternal education, number of prenatal consultations and the use of pacifier the previous day

^h^Association adjusted for maternal education, mother’s marital status, household food security situation, primiparity, use of pacifier the previous day and use of baby bottle the previous day

^i^Association adjusted for maternal education, mother’s marital status, mother’s age group, primiparity, use of pacifier the previous day and use of baby bottle the previous day

^j^Association adjusted for maternal education, household food security situation, mother’s age group and use of pacifier the previous day

^k^Association adjusted for primiparity

^l^Association adjusted for maternal education, mother’s work situation, number of prenatal consultations and type of child birth

^m^Association adjusted for mother’s marital status, mother’s work situation, number of prenatal consultations, primiparity, use of pacifier the previous day and hospitalization of the child in the last year

## Discussion

We found a low prevalence of minimum dietary diversity and a high consumption of UPF, especially yogurts, cookies or crackers and foods or beverages with added sugar, honey or artificial sweetener. Furthermore, the breastmilk intake in the second year of life was associated with lower odds of consuming UPF and sweetened beverages.

Along with breastfeeding, complementary feeding contribute to the child’s full growth and development. A healthy, responsive and opportune complementary feeding is related to adequate eating habits establishment, and to child’s protection child against a high energy intake and consequently against overweight and obesity [1]. In our study, the introduction of complementary feeding was considered untimely – too early - for 38.3% of the children under 6 months. In addition, we observed an elevated prevalence of UPF consumption for all children. These results are similar to other studies that evaluated early childhood food consumption in Brazil [10, 25]. UPF consumption in the first 2 years of life is not recommended because they normally present an elevated energy density and high levels of sugars, fat, sodium and additives, they are hyper palatable, induce the child to frequent consumption and negatively influence future food preferences. The UPF consumption is also related to higher prevalence of obesity, chronic diseases and nutritional deficiencies in the first years of life and may also compromise healthy foods consumption, associated with the adequate child growth and development [1, 12, 13, 26].

The prevalence of minimum dietary diversity was higher than that verified in previous Brazilian studies [27, 28], however, it is still considered very low and may reflect in micronutrient deficiencies [8]. Nevertheless, it is necessary to expound this indicator carefully regarding children aged 6–12 months. Complementary feeding is introduced gradually, may not be completely established between 6 and 8 months and breastmilk can be still the main nutrients source and contribute up to two-thirds of children energy intake in the first months of food introduction [29, 30]. With regard to healthy feeding indicators, most children consumed foods rich in iron and vitamin A and its consumption should continue to be encouraged. Micronutrient deficiencies, especially anemia and hypovitaminosis A, are still important factors for high morbidity rates among children under 5 years old in Brazil and other middle-income countries [31].

Although surprising, our study did not show an association between breastmilk intake and healthy feeding indicators. Several studies show that the exposure to a greater variety of flavors, during breastfeeding, can facilitate the acceptance of new foods during complementary food introduction and it is also expected that breastfeeding mothers will be more likely to encourage healthy foods consumption [1, 14, 15]. Still, some studies relate healthy feeding indicators to other socioeconomic factors, including income and education [4, 32], which might play a more important role in our study when compared to breastmilk intake.

The breastmilk intake was associated with lower odds of consuming UPF by children. Some studies reported that breastmilk intake is related to lower consumption of UPF and of sweetened beverages among children between 6 and 12 months [10, 15, 19]. Our results brings unprecedented data about breastmilk as a long-term protection factor for UPF consumption in the first 2 years, not investigated in previous studies. For children who were breastfed in the second year of life, in addition to breastfeeding’s own benefits, it was also associated with a lower consumption of sweetened beverages, which translates into better dietary patterns and can prevent obesity [26]. Furthermore, among other factors, family’s consumption behavior can mediate children feeding practices, so it can enhance healthy or unhealthy feeding patterns. In addition, mothers who breastfeed tend to limit the exposure and consumption of unhealthy foods by their children [1], suggesting greater health literacy relating to optimal nutrition.

It is important to highlight that personal, familial, social, economic and structural factors are associated with breastfeeding practice and food consumption behavior. Those factors can determinate the mother’s possibility to breastfed, for how long breastfeed their children and to provide adequate and healthy complementary feeding. Those factors are also associated with the family’s opportunities to have healthy feeding patterns and to provide a nurturing and a healthy environment for their children. Better chances of healthy feeding practices are usually associated with family income, mother’s educational level, adequate working conditions and maternity leave, and family and community support as well [33, 34].

While our study contributes to the strengthening of the evidence on the influence of breastmilk on complementary feeding, it has some limitations. First, the cross-sectional design does not allow the establishment of causality between breastmilk intake and the outcomes. In addition, the food consumption investigation, conducted the day before the interview, is not able to evaluate the usual intake or the food consumption frequency. However, this technique allows the reduction of memory bias, is easy to apply and is used for monitoring and evaluating food indicators in Brazil within the scope of Primary Health Care [22]. Regarding the sample, we included only children treated in Primary Health Care Units, however, in Federal District, Primary Health Care of Unified Health System (SUS) covers 61.26% of the local population [35].

## Conclusions

In conclusion, as we found that breastmilk intake in the second year of life was associated with lower odds of consuming UPF and sweetened beverages, strategies to scale up breastfeeding have great potential to prevent lifetime health problems. Likewise, it is also important to invest in cost-effective actions targeting the reduction of UPF consumption by children under 2 years old. However, these actions are not simple and require multi-sectorial mobilization and engagement. In order to develop a supportive environment for breastfeeding and healthy eating habits over time, interventions on structural, contextual and individual determinants are necessary, including the formulation of public policies at the national level, local coordination for their implementation, and health professional training for counseling and support of mothers [2].

## Supplementary information

**Additional file 1.** Dataset

## Data Availability

The dataset supporting the conclusions of this article is included within the article (Additional file 1).

## Abbreviations

EBIA: Brazilian Household Food Insecurity Measurement Scale
PHU: Primary Health Units
SD: Standard deviation
UPF: Ultra-processed foods (UPF)
